# Cannabis poisonings in Australia following the legalisation of medicinal cannabis, 2014–24: analysis of NSW Poisons Information Centre data

**DOI:** 10.5694/mja2.52586

**Published:** 2025-01-23

**Authors:** Rose Cairns, Sara Allaf, Nicholas A Buckley

**Affiliations:** ^1^ The University of Sydney Sydney NSW; ^2^ NSW Poisons Information Centre Children's Hospital at Westmead Sydney NSW

**Keywords:** Poisoning, Toxicology, Poison control centers, Drug overdose, Drug misuse, Legislation, drug, Policy, drugs and alcohol, Street drugs, Psychotropic drugs

Several countries have legalised medicinal cannabis during the past two decades; more recently, some jurisdictions, including parts of the United States, Canada, and Uruguay, have legalised recreational cannabis.^1^ Legislative models differ between countries, particularly with regard to access to medicinal cannabis, commercialisation, the availability of cannabis dispensaries, and cannabis‐containing confectionery (“edibles”).^2^

One potential harm of increased access to cannabis is poisoning. It is widely believed that cannabis is safe in overdose,^3^ but it can cause central nervous system (CNS) excitation, CNS depression, hallucinations, psychosis, and cardiac dysrhythmias.^4^ The risk of severe toxicity is greater for children, in whom it can lead to apnoea and coma; in one United States study, 32 of 60 children (0–10 years) hospitalised with cannabis intoxication required intensive care.^5^ Several studies have reported increases in the number of poisonings following medicinal and recreational cannabis legalisation, particularly in children.^6^ Edibles are particularly high risk products because of their palatability and the possibility of large ingestions.^2^ Most reports on this problem are from North America.^6^

In Australia, the medicinal use of cannabidiol (CBD) was legalised in June 2015, and that of cannabis and tetrahydrocannabinol (THC) in November 2016.^7^ We therefore evaluated recent cannabis poisoning exposures in Australia, stratified by ingestion intent, age group, and product type. We analysed data from the New South Wales Poisons Information Centre (NSWPIC), which receives about 50% of all calls to Australian poisons information centres; 65% of calls are from within NSW, 35% from other states.^8^ We extracted data on demographic and exposure characteristics, patient disposition, and cannabinoid product types for calls during 1 July 2014 – 30 June 2024. We calculated crude and age‐adjusted population exposure call rates (Supporting Information, supplementary methods), and used Joinpoint regression (version 4.9.0.1) to estimate annual percentage changes (APCs) in age‐adjusted rates and to detect trend change points. The study was approved by the Sydney Children's Hospitals Network Human Research Ethics Committee (2021/ETH00165).

There were 3796 calls about cannabis poisoning exposures (2039 regarding exposures of boys or men, 54%) during 2014–24. The exposed person exhibited symptoms of poisoning at the time of the call in 3184 cases (84% of calls); 2783 people (74%) were in hospital at the time of the call or were referred to hospital (Supporting Information, table 1). The number of calls increased during 2014–24 by 12.8% per year (95% confidence interval [CI], 10.3–15.4% per year), and no trend change points were detected (Box 1). Intentional cannabis exposures were reported by 2981 calls (79% of calls), and the number increased by 9.2% (95% CI, 6.3–12.2%) per year. Unintentional cannabis exposures were reported by 815 calls (21%), and the number increased by 30.0% (95% CI, 23.5–36.8%) per year (Box 1, Box 2).

Box 1 Calls to the New South Wales Poisons Information Centre regarding cannabinoid exposures, 1 July 2014 – 30 June 2024: crude and age‐adjusted rates*

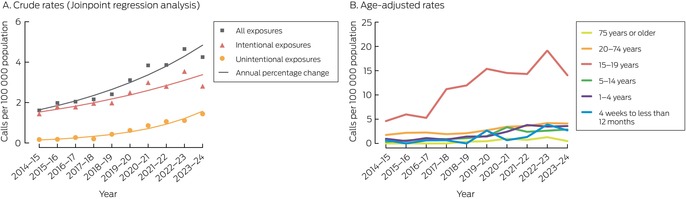

* The raw data (call numbers) are depicted in the Supporting Information, figure 1.

Box 2 Calls to the New South Wales Poisons Information Centre regarding cannabinoid exposures, 1 July 2014 – 30 June 2024, by exposure intention and cannabis product type*

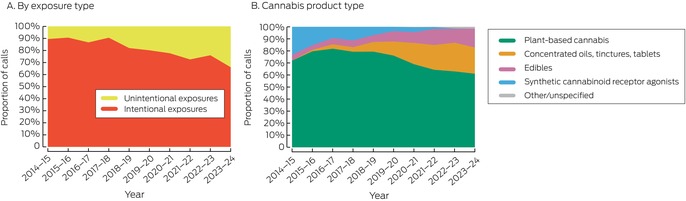

* Intentional exposures include recreational exposure, deliberate self‐poisoning, and other intentional misuse; unintentional exposures include therapeutic errors, accidental exploratory exposures, and adverse reactions.

The age‐adjusted cannabis poisoning exposure rate was highest for adolescents (15–19 years; 11.4 calls per 100 000 population per year); the age‐adjusted rate for unintentional exposures was highest for toddlers (1–4 years; 1.9 calls per 100 000 population per year) (Supporting Information, table 2).

Plant‐based cannabis (ie, flower or leaf) was the reported exposure form in 2663 cases (70% of all cases) (Supporting Information, table 1); the proportion declined from 72% in 2014–15 to 61% in 2023–24, and the proportions of cases involving concentrates (from 0 to 124 calls, 22%) and edibles increased (from nine calls, 5%, to 88 calls, 16%) (Box 2). Across the decade, concentrates (320 calls, 39%), plant‐based cannabis (302 calls, 37%), and edibles (171 calls, 21%) were the three product forms most frequently involved in unintentional exposure calls (Supporting Information, table 1). Calls about exposures to edibles have increased rapidly since 2019–20, particularly gummy or lolly forms (89 cases, all reported since 2019–20) (Supporting Information, figure 2).

The number of calls to NSWPIC regarding cannabis poisoning increased significantly during 2014–24, but we found no trend change points, including during the 2015–16 re‐scheduling of medicinal use products. Most prescribed cannabis use in Australia involves unapproved products permitted under the Special Access Scheme B (SAS‐B).^9^ Administrative hurdles may have prevented the rapid uptake of medicinal cannabis immediately after its legalisation; few SAS‐B approvals were recorded during 2016–18, but the 2018 launch of a streamlined SAS‐B portal system was followed by a dramatic increase in approval numbers.^9^ Further, a 2019 survey found that only 3.9% of Australians who used cannabis for medicinal purposes obtained it on prescription.^10^ The increase in exposure calls could therefore reflect a steady increase in cannabis use during 2014–24 because of changing social norms and perceptions of cannabis safety and legality. The more rapid increase in calls about exposures in young people could reflect the increasing availability of edible forms, which can be medically prescribed or obtained illicitly. This interpretation would be consistent with survey findings that about 15% of Australians who used cannabis for medicinal purposes used oral formulations in 2016 (ie, prior to its legalisation),^11^ but 33% did so in 2022–23.^12^

Limitations of our study include the fact that NSWPIC does not routinely undertake follow‐up enquiries; final outcomes are consequently unknown. Information about the cannabis form is as reported by the caller, and was sometimes missing. Some exposures were to multiple substances, and symptoms and dispositions could therefore be related to substances other than cannabis. As the incidence of cannabinoid poisoning is relatively low, the number of calls was small. Further, we used Joinpoint to detect change points rather than for hypothesis testing; the low call numbers prior to 2016 limited the power of our analysis to detect statistically significant changes following re‐scheduling.

We found that the number of cannabis poisonings reported increased significantly in Australia during 2014–24, particularly exposures of children and adolescents. The reported number of exposures to edibles, which pose a particular risk for young children,^13^ has increased. Our findings are relevant to discussions of increasing access to medicinal cannabis and legalising its recreational use. Lessons learned overseas with different legislative models could be applied in Australia. For example, the sale of edibles is not permitted in some Canadian provinces, and significantly more children are hospitalised with cannabis intoxication in provinces where they are sold.^2^ While using orally ingested cannabis forms may be less harmful in the long term than smoking cannabis, the acute poisoning risk posed by edible forms of cannabis must be considered. Particular caution needs to be applied to confectionery forms that are attractive for children.

## Open access

Open access publishing facilitated by the University of Sydney, as part of the Wiley – the University of Sydney agreement via the Council of Australian University Librarians.

## Competing interests

Rose Cairns has received honoraria and speaker fees from the Pharmacy Guild of Australia and Reckitt, and an untied educational grant from Reckitt (all unrelated to this article).

## Data sharing

For privacy reasons, NSWPIC data cannot be shared without prior ethics approval.

## Supporting information


Supplementary methods and results
