# Supplementary material for: Cannabis poisonings in Australia following the legalisation of medicinal cannabis, 2014–24: analysis of NSW Poisons Information Centre data
Source: Med J Aust. 2025 Jan 23;222(3):155–7. doi: 10.5694/mja2.52586 (PMC11830224; doi:10.5694/mja2.52586)
Supplement: Supplementary file 1 — Supplementary methods and results [file MJA2-222-155-s001.pdf]

MJA
The Medical Journal of Australia

Supporting Information

Supplementary methods and results

This appendix was part of the submitted manuscript and has been peer reviewed.
It is posted as supplied by the authors.

Appendix to: Cairns R, Allaf S, Buckley NA. Cannabis poisonings in Australia following the legalisation of medicinal cannabis, 2014–24: analysis of NSW Poisons Information Centre data. *Med J Aust* 2025; doi: 10.5694/mja2.52586.

Supplementary methods

Calculation of population rates

We calculated crude population-adjusted rates by dividing exposure call counts by Australian Bureau of Statistics (ABS) mid-year population estimates [1]. For age-adjusted rates, we used the Australian estimated resident population by single year of age data [1], summed to align with New South Wales Poisons Information Centre (NSWPIC) database age categories (less than one year, 1 to 4 years, 5 to 14 years, 15 to 19 years, 20 to 74 years, 75 years or older). ABS estimates were divided by two to account for the population coverage of NSWPIC (50% of all calls to poisons information centres in Australia).

Supplementary results

Table 1. Characteristics of calls to the New South Wales Poisons Information Centre (NSWPIC) regarding cannabis and cannabinoid exposures, 1 July 2014 – 30 June 2024

Characteristic	Intentional exposures†	Unintentional exposures‡	Total
**Total**	**2981**	**815**	**3796**
**Sex***			
Male	1619 (54%)	420 (52%)	2039 (54%)
Female	1340 (45%)	376 (46%)	1716 (45%)
Unknown	22 (1%)	19 (2%)	41 (1%)
**Age category**			
Infant (4 weeks to less than 1 year)	§	§	20 (<1%)
Toddler (1 to 4 years)	§	§	120 (3%)
Child (5 to 14 years)	183 (6%)	84 (10%)	267 (7%)
Adolescent (15 to 19 years)	829 (28%)	49 (6%)	878 (23%)
Adult (20 to 74 years)	1946 (65%)	509 (62%)	2455 (65%)
Older adult (75 years or older)	7 (<1%)	34 (4%)	41 (1%)
Unknown	10 (<1%)	5 (1%)	15 (<1%)
**Disposition/handling**			
Stay at home	520 (17%)	211 (26%)	731 (19%)
Referral to general practitioner	40 (1%)	33 (4%)	73 (2%)
At general practitioner surgery	§	§	6 (<1%)
Hospital referral	658 (22%)	238 (29%)	896 (24%)
In hospital	1622 (54%)	265 (33%)	1887 (50%)
Other	130 (4%)	66 (8%)	196 (5%)
Unknown	5 (<1%)	0	5 (<1%)
**Cannabinoid category type**			
Plant-based cannabis (illicit or prescribed)	2361 (79%)	302 (37%)	2663 (70%)
Concentrated oils, tinctures, tablets/capsules	207 (7%)	320 (39%)	527 (14%)
Edibles	194 (7%)	171 (21%)	365 (10%)
Synthetic cannabinoid receptor agonists	188 (6%)	11 (1%)	199 (5%)
Vape liquid	15 (1%)	7 (1%)	22 (1%)
Other/unknown	§	§	20 (1%)
**Cannabinoid content, if specified**			
Delta-9-tetrahydrocannibinol (THC)	98 (52%)	108 (34%)	205 (40%)
Cannabidiol (CBD)	52 (28%)	119 (37%)	171 (34%)
THC/CBD blend	33 (18%)	93 (29%)	126 (25%)
Δ-8-THC (alone or in blends)	§	§	6 (<1%)
**Symptoms at time of call**			
Asymptomatic	266 (9%)	186 (23%)	452 (12%)
Symptomatic, related to exposure	2347 (79%)	450 (57%)	2797 (74%)
Symptomatic, deemed unrelated to exposure	51 (2%)	20 (2%)	71 (2%)
Symptomatic, not known if related	248 (8%)	139 (17%)	387 (10%)
Unknown symptoms	68 (2%)	20 (4%)	88 (2%)

* The NSWPIC data field for "sex" records the sex or gender reported by the caller.

† Recreational exposures, deliberate self-poisoning, and other intentional misuse.

‡ Unintentional poisonings include therapeutic errors, accidental exploratory exposures, adverse reactions.

§ Counts of less than five or allowing calculation of cell counts of less than five suppressed.

Figure 1. Calls to the New South Wales Poisons Information Centre regarding cannabis and cannabinoid exposures, 1 July 2014 – 30 June 2024. A. Call numbers (Joinpoint regression analysis). B. Calls by age group

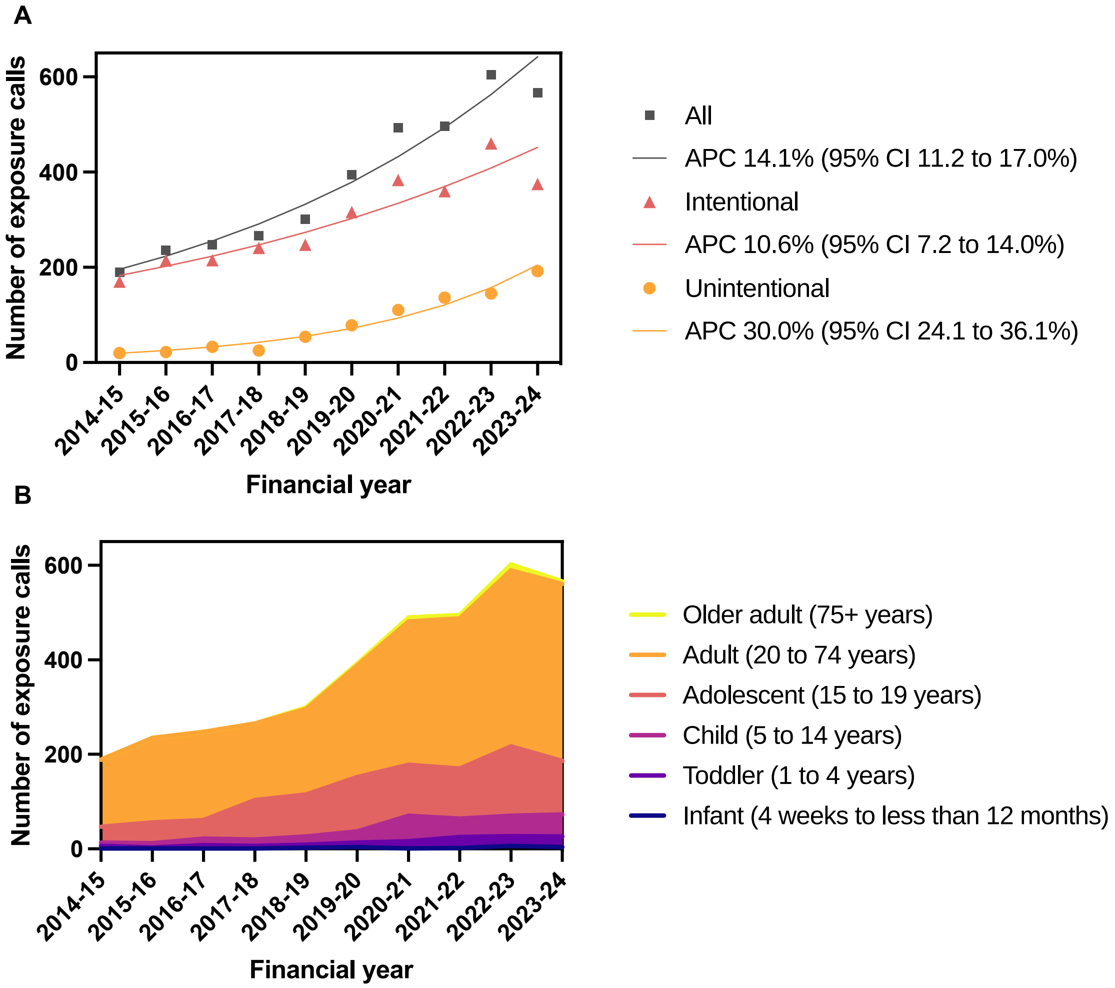

APC = annual percentage change.

Table 2. Estimated population-adjusted rates of calls to the New South Wales Poisons Information Centre (NSWPIC) regarding cannabis and cannabinoid exposures, , 1 July 2014 – 30 June 2024, by age category and intent

		Unintentional exposure calls		Intentional exposure calls		All exposure calls	
**Age group**	**Estimated population covered by NSWPIC***	**Number**	**Rate (per 100,000/year)**	**Number**	**Rate (per 100,000/year)**	**Number**	**Rate (per 100,000/year)**
Infants (4 weeks to less than 12 months)	153,910	17	1.1	3	0.19	20	1.3
Toddlers (1-4 years)	602,627	117	1.94	3	0.05	120	1.99
Children (5-14 years)	1,625,444	84	0.52	183	1.13	267	1.64
Adolescents (15-19 years)	766,960	49	0.64	829	10.8	878	11.4
Adults (20-74 years)	8,865,512	509	0.57	1946	2.2	2455	2.77
Older adults (75 years or older)	992,748	34	0.34	7	0.07	41	0.41

* Population adjustment used Australian Bureau of Statistics estimated resident population by age as of 30 June 2022 [1]. Australian population was divided by two to account for the fact that New South Wales Poisons Information Centre takes 50% of poisoning calls. Overall rate was divided by 10 to estimate the annual rate over the 10-year study period.

Figure 2. Calls to the New South Wales Poisons Information Centre regarding edible cannabinoid exposures, 1 July 2014 – 30 June 2024.* A. Call numbers. B. Population-adjusted rates†

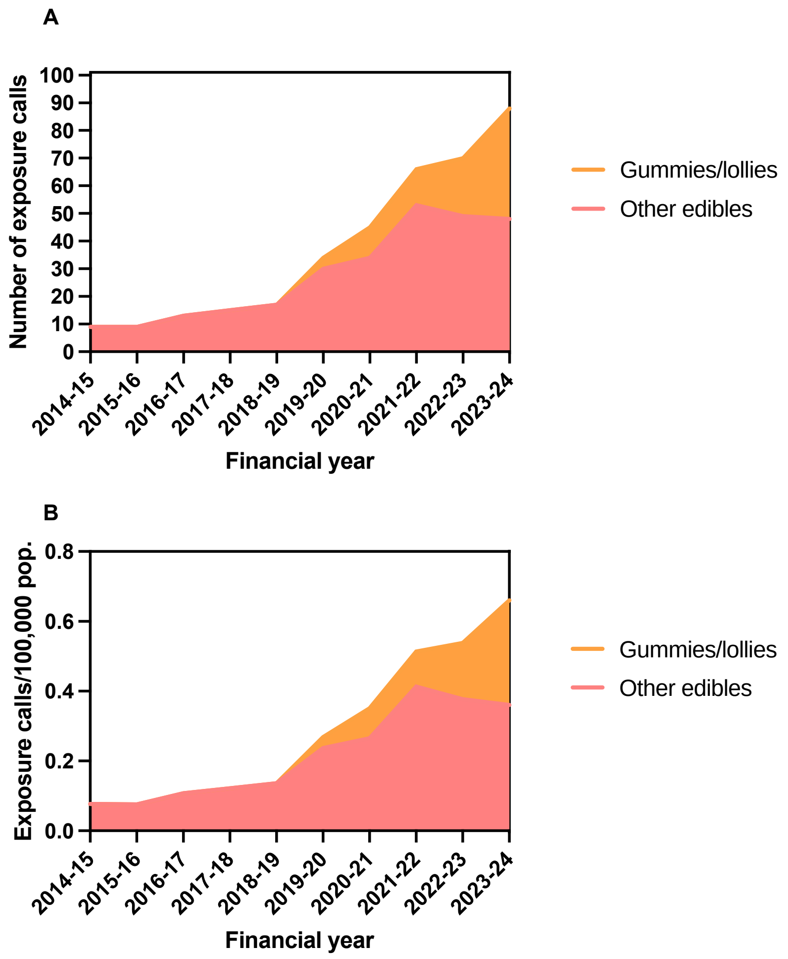

* "Other edibles" includes cookies, brownies, and other miscellaneous forms of food-based cannabis products.
† Based on Australian Bureau of Statistics mid-year population estimates [1].

Reference

1. Australian Bureau of Statistics. National, state and territory population, December 2023. 13 June 2024. https://www.abs.gov.au/statistics/people/population/national-state-and-territory-population/dec-2023 (viewed June 2024).
